# Coronavirus Diversity in an Italian Bat Population Sampled During the Pandemic in 2020

**DOI:** 10.3390/ani16203319

**Published:** 2026-10-21

**Authors:** Gabriele Vaccari, Ilaria Di Bartolo, Luca Dorigo, Angelo Leandro Dreon, Stefano Toppo, Margherita Morpurgo, Edoardo Vernier, Luigi Bertolotti, Andrea Crisanti, Maurizio Guido Paoletti, Enrico Lavezzo, Luca De Sabato

**Affiliations:** 1 Department of Food Safety, Nutrition and Veterinary Public Health, Istituto Superiore di Sanità, 00161 Rome, Italy; gabriele.vaccari@iss.it (G.V.); ilaria.dibartolo@iss.it (I.D.B.); luca.desabato@iss.it (L.D.S.); 2 Friulian Museum of Natural History, 33100 Udine, Italy; luca.dorigo@comune.udine.it; 3 Independent Researcher, 33080 Frisanco, Italy; leandro.dreon@gmail.com; 4 Department of Molecular Medicine, University of Padova, 35131 Padova, Italy; stefano.toppo@unipd.it (S.T.); andrea.crisanti@unipd.it (A.C.); 5 Department of Pharmaceutical and Pharmacological Sciences, University of Padova, 35131 Padova, Italy; margherita.morpurgo@unipd.it; 6 Museum of Nature and Humankind, University of Padova, 35137 Padova, Italy; e.vernier@libero.it; 7 Department of Veterinary Sciences, University of Torino, 10095 Torino, Italy; luigi.bertolotti@unito.it; 8 Department of Biology, University of Padova, 35121 Padova, Italy

**Keywords:** bats, Alphacoronavirus, Betacoronavirus, Europe, SARS-CoV-related

## Abstract

Evolutionary studies have established a correlation between human and bat coronaviruses (CoV), identifying bats as the natural reservoir for *Coronaviridae*. Over the past two decades, three major zoonotic spillover events have been described: Severe Acute Respiratory Syndrome CoV (SARS-CoV) and Middle East Respiratory Syndrome CoV (MERS-CoV), which caused significant global outbreaks, and SARS-CoV-2, which triggered the COVID-19 pandemic. Despite these events, the true diversity of coronavirus species harbored by bats remains largely unmapped, even as the discovery of novel strains continues to accelerate. In this study, we analyzed 37 fecal swab samples collected in Northern Italy, targeting the bat genera *Miniopterus*, *Myotis*, and *Rhinolophus.* Coronaviruses were detected using a pan-coronavirus (Pan-CoV) nested RT-PCR in five bat samples: three tested positive for Alphacoronaviruses (Alpha-CoVs) and two for Betacoronaviruses (Beta-CoVs). Positive samples were subjected to Pan-CoV Illumina next-generation sequencing (NGS), and two samples yielded sufficient read depth for robust contig assembly. Phylogenetic analysis revealed the co-circulation of three distinct Alpha-CoVs within a *Miniopterus schreibersii (M. schreibersii)* fecal sample and the presence of a SARS-CoV-related strain in a *Rhinolophus hipposideros* (*R. hipposideros*) fecal sample. Both the Alpha-CoVs and the Beta-CoVs were most closely related to European strains. These findings confirm the widespread circulation of diverse CoVs in Italian bats and underscore the need for continued molecular epidemiological surveillance to unravel the full extent of CoV heterogeneity in European wildlife.

## Simple Summary

Bats act as natural reservoirs for many coronaviruses, harboring these pathogens asymptomatically. Owing to the emergence of zoonotic coronaviruses over the past 25 years, monitoring them directly in bat populations is critical. Identifying viruses in wildlife allows researchers to detect strains that could potentially spill over to humans and trigger future outbreaks before they occur. Tracking wildlife near human habitats reveals where human–animal interactions create potential disease hotspots. In addition, mapping current viral diversity allows scientists to design treatments ahead of time, rather than reacting after a new threat emerges. In short, searching for viruses at their source may allow us to anticipate pandemics rather than merely respond to them.

## 1. Introduction

Bats are globally recognized as major reservoirs of a wide range of coronaviruses. Their ecological diversity, social behavior and long-term virus–host coevolution contribute to the maintenance and circulation of highly diverse coronavirus populations [1].

Coronaviruses (order *Nidovirales*, family *Coronaviridae*, subfamily *Orthocoronavirinae*) are enveloped viruses characterized by a positive-sense single-stranded RNA genome, which encodes the polyprotein ORF1ab alongside structural and accessory proteins. Strains capable of infecting mammals are primarily found within the genera *Alphacoronavirus* (*Alpha-CoV*) and *Betacoronavirus* (*Beta-CoV*) [2].

Over the past 25 years, three highly pathogenic Beta-CoVs have emerged: Severe Acute Respiratory Syndrome coronavirus (SARS-CoV), Middle East Respiratory Syndrome coronavirus (MERS-CoV) and, most recently, SARS-CoV-2, which caused the COVID-19 pandemic, resulting in over 7 million confirmed deaths worldwide and continuing to circulate globally (https://covid19.who.int/, accessed on 6 July 2026). These CoVs are believed to have originated from bat-associated coronavirus lineages and emerged in humans through zoonotic spillover events involving intermediate hosts. These events have highlighted the importance of characterizing coronavirus diversity in bat populations and identifying viral lineages with zoonotic potential. Since coronaviruses are characterized by frequent recombination and a high mutation rate [3,4], these viruses may continue to evolve, potentially leading to the emergence of novel variants characterized by higher pathogenicity, increased transmissibility, or resistance to existing vaccines and therapeutics.

Despite extensive documentation of bat-borne CoVs in both anthropogenic and natural environments, a comprehensive global characterization of the infection landscape is still lacking. In Europe, over 30 studies have evaluated CoV prevalence in bats. To date, CoVs have been identified in nine genera belonging to the families Rhinolophidae, Vespertilionidae, Molossidae and Miniopteridae: *Rhinolophus*, *Myotis*, *Pipistrellus*, *Plecotus*, *Nyctalus*, *Hypsugo*, *Eptesicus*, *Tadarida* and *Miniopterus*. These represent approximately half of the bat species found in Europe [5]. Interestingly, although European strains are diverse, some studies, for example from Germany [6], have reported unexpected genomic similarities with Chinese strains. This similarity, despite the considerable geographic separation, points to the role of shared ecological niches, such as specific forest types and cave microclimates, in shaping viral evolution among bat populations worldwide.

In Italy, Alpha-CoVs have been reported in bats of the families Vespertilionidae (*Pipistrellus kuhlii*, *Pipistrellus pipistrellus*, *Myotis myotis*, *Myotis blythii*/*Myotis oxygnathus*, *Myotis nattereri*), Miniopteridae (*Miniopterus schreibersii*; subgenus *Minunacovirus*) and Rhinolophidae (*Rhinolophus ferrumequinum*) [7,8,9,10,11,12,13], whereas Beta-CoVs have been detected in Rhinolophidae (*Rhinolophus ferrumequinum*, *Rhinolophus hipposideros*; subgenus *Sarbecovirus*) [7,9,11,14,15], in Vespertilionidae, either MERS-CoV-related (*Merbecovirus*: *Pipistrellus kuhlii*, *Hypsugo savii*, *Nyctalus noctula*, *Eptesicus serotinus*) or SARS-CoV-related. (*Plecotus auritus*) [7,8,9,15,16], and in Molossidae (*Tadarida teniotis*; SARS-CoV-related) [15].

Despite increasing knowledge on bat coronaviruses in Europe, genomic information remains limited for many geographic areas, including Italy. During the first wave of the COVID-19 pandemic, bats from Northern Italy were screened for coronaviruses using molecular and sequencing approaches to investigate coronavirus diversity and characterize the circulating Alpha-CoV and Beta-CoV lineages. This survey identified both Alpha-CoVs and SARS-CoV-related Beta-CoVs, and resulted in the recovery of near-complete viral genomes and in the detection of multiple Alpha-CoV lineages within a single sample.

## 2. Materials and Methods

### 2.1. Sampling

**Figure 1:**
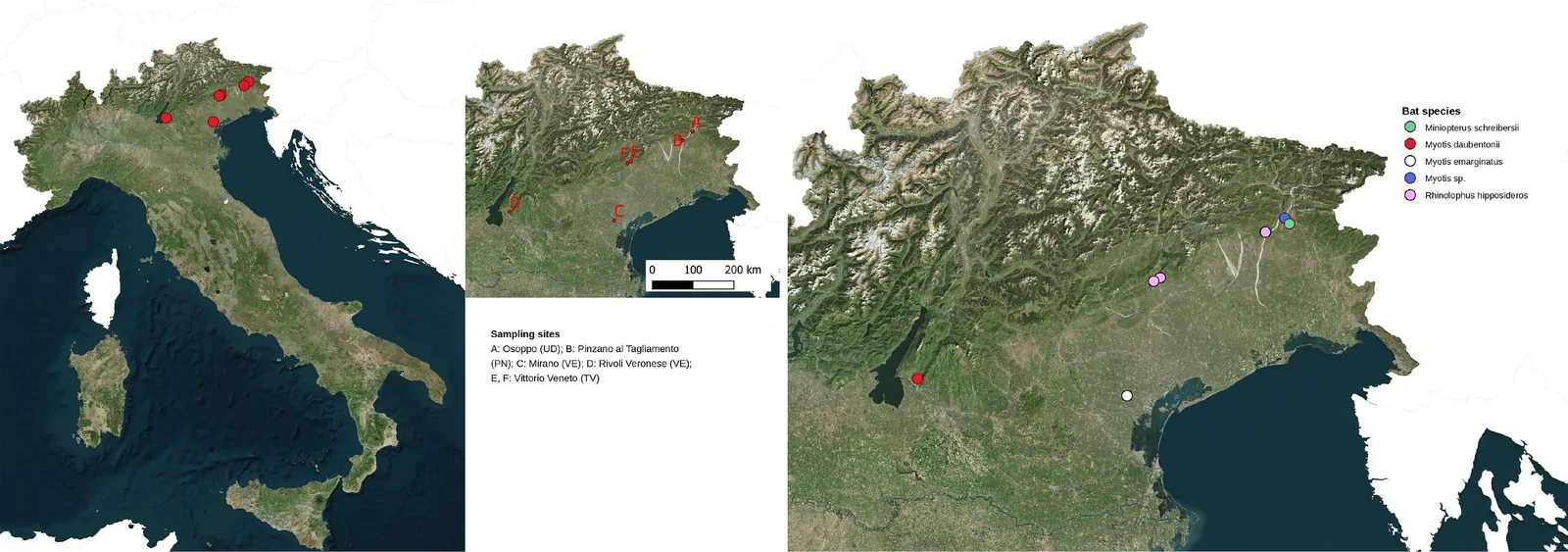
Sampling sites and identified bat species.

In June 2020, bat fecal samples were collected from maternity colonies in north-eastern Italy (Veneto and Friuli Venezia Giulia regions; Figure 1) as part of a survey aimed at detecting coronaviruses in bat populations. The five sampled roosts (Supplementary Table S1) include some of the most important known maternity colonies in the study area and host species belonging to the genera *Myotis*, *Rhinolophus* and *Miniopterus*, allowing the investigation of coronavirus circulation across different bat species and colony types.

Sampling was preferentially conducted in monospecific maternity colonies whose species identity had been previously confirmed through direct observation and long-term monitoring. Where possible, sterile polyethylene sheets were placed beneath roosting clusters, and freshly deposited droppings were collected from the sheets using sterile flocked swabs with transport medium (eSwab, Copan Italia S.p.A., Brescia, Italy).

One roost (OS) hosted a mixed-species maternity colony, in which a cluster of *Myotis myotis (M. myotis)* and *Myotis blythii (M. blythii)* coexisted with a numerically dominant population of *M. schreibersii*. Here, separate sheets were positioned beneath the main *M. schreibersii* aggregation and beneath the *Myotis* spp. cluster, and fecal pellets were collected separately. In this roost, host attribution of the samples was based on the position of the feces relative to the clusters and on the size and morphology of the pellets, which differed between the two groups; no molecular identification of the host species was performed. The distinction was based on published descriptions reporting generally larger pellet dimensions, and in particular a larger diameter, in *Myotis* spp. than in *M. schreibersii* [17]. Because the droppings were collected non-invasively, each sample consists of freshly deposited fecal material that cannot be attributed with certainty to an individual bat. Accordingly, throughout this study the sampling unit is the fecal sample rather than the individual animal.

Field operators wore personal protective equipment (PPE), including FFP2 respirators and disposable latex gloves, which were replaced after each sample to prevent cross-contamination. Samples were kept at low temperature during storage and transport until delivery to the laboratories of the University of Padua for analysis.

In total, 37 fecal samples were collected: 20 from *R. hipposideros*, 7 from *Myotis emarginatus (M. emarginatus)*, 2 from *Myotis daubentonii (M. daubentonii)*, 4 from cf. *M. schreibersii* and 4 from cf. *Myotis* spp. (Supplementary Table S1).

### 2.2. RNA CoV Screening

Total RNA was extracted from 150 μL of sample using the QIAamp Viral RNA Mini Kit (Qiagen, Milan, Italy). The RNA was eluted in 100 μL of RNase-free water and stored at −80 °C. SARS-CoV-2 and pan-Sarbecovirus screening was performed by real-time RT-PCR [18]. Samples were also screened by a Pan-CoV nested RT-PCR, targeting a short genomic region within the RNA-dependent RNA polymerase (RdRp) and amplifying approximately 440 bp [19].

### 2.3. Whole-Genome Sequencing

Sequencing libraries were prepared using Illumina RNA Prep with Enrichment (L) Tagmentation (Illumina, Milan, Italy) in combination with the Pan-Coronavirus Panel (Illumina) and Illumina DNA/RNA Unique dual (UD) Indexes, following the manufacturer’s instructions. Briefly, 10 µL of total RNA per sample were reverse transcribed and converted into double-stranded cDNA, which was then tagmented using bead-linked transposomes and amplified in a limited-cycle PCR that introduced unique dual indexes, allowing unambiguous demultiplexing and minimizing index hopping between multiplexed samples. Amplified libraries were pooled and enriched with the Pan-Coronavirus Panel, a hybrid-capture assay comprising biotinylated oligonucleotide probes tiled across the reference genomes of 225 known human and animal coronaviruses, including 69 Alphacoronavirus and 59 Betacoronavirus genomes, and covering a broad range of animal hosts, with bat-derived genomes being the most represented (*n* = 64). Hybrid-capture target enrichment allows sensitive detection of viral targets. Probe-bound fragments were captured on streptavidin magnetic beads, eluted and amplified to generate the final enriched libraries. Enriched libraries were sequenced on an Illumina NextSeq 2000 instrument using a P1 flow cell in a 2 × 150 bp paired-end configuration, targeting a minimum of 1 million reads per sample, in accordance with the read length and sequencing depth recommended for this panel. Base calling and demultiplexing were performed on-instrument, and the resulting per-sample FASTQ files were used for the downstream analyses described in Section 2.4.

### 2.4. Bioinformatic Analysis

To obtain a high-quality consensus sequence, we employed an integrated approach:

(1) Quality check and trimming: raw reads (FASTQ files) were trimmed using Trimmomatic v0.39 [20] with the following parameters: LEADING:3, TRAILING:3, SLIDINGWINDOW:4:15, and MINLEN:50, retaining reads ≥ 50 bp.
(2) De novo assembly: cleaned and trimmed reads were assembled using SPAdes v3.15.5 [21] with k-mer lengths of 21, 33, 55, 77, 99 and 127 and a minimum contig length of 500 bp, in order to identify the viral sequences present in the samples.
(3) Reference selection: the resulting contigs were compared against the NCBI database using blastn to select the most similar coronavirus reference genome based on an E-value threshold < 1 × 10^−5^ and minimum query coverage > 80.0%.
(4) Hybrid genome construction: selected contigs from the de novo assembly were used to improve the reference genome sequence by targeted replacement. Each contig was aligned to the reference genome using the blastn output coordinates. Contigs were then substituted in place of the corresponding reference sequence regions based on their alignment coordinates. Sequence gaps between adjacent contigs were temporarily bridged with the corresponding reference genome sequence for the sole purpose of obtaining a continuous scaffold. It should be emphasized that this hybrid sequence was used exclusively as a scaffold for the subsequent read-mapping step and does not correspond to the consensus genomes reported in this study. This hybrid approach integrates high-confidence sequence information from de novo assembly of our sample with the conserved architecture and complete gene annotations of the reference coronavirus genome, providing a continuous template onto which the sequencing reads were subsequently mapped.
(5) Mapping and final consensus: the original reads were mapped onto the hybrid genome using Bowtie2 [22] and visualized with the IGV tool [23]. The final consensus sequence was generated using the iVar consensus tool with the following criteria: bases with a read depth < 10× were marked as ambiguous (N), and consensus bases were called where ≥80.0% of mapped reads supported the same nucleotide. Consequently, every position not supported by at least 10× coverage of the reads generated in this study, including the reference-derived regions used to bridge contig gaps within the scaffold, was reported as N, so that no reference-derived nucleotide was retained in the final sequences. The consensus sequences deposited in GenBank therefore contain exclusively nucleotides called from the reads produced in this study.

### 2.5. Phylogenetic and Sequence Analyses

For the phylogenetic analysis, three datasets were built: two based on complete genomes and one based on RdRp sequences. Dataset A included 39 complete genomes: 3 nearly complete Alpha-CoV genomes from this study, 31 Alpha-CoV reference complete genomes downloaded from NCBI RefSeq database, 3 complete genomes most closely related to the sequences from this study, downloaded from NCBI nucleotide database, and 2 sequences from Beta-CoV as an outgroup (NC_045512, NC_004718). Dataset B included 47 complete genomes: 1 Beta-CoV sequence from this study, 3 Sarbecovirus (Beta-CoVs) reference complete genomes downloaded from NCBI RefSeq database, 2 complete genomes most closely related to the sequences from this study and 39 SARS-CoV-related sequences downloaded from NCBI nucleotide database, and 2 sequences from Merbecovirus as an outgroup (NC_019843, NC_038294). Dataset C included 89 sequences from RdRp region (395 nt in length): 7 CoV sequences from this study (5 Alpha-CoV and 2 Beta-CoV), 49 reference sequences downloaded from NCBI RefSeq database, 33 representative sequences reported in bats worldwide and downloaded from NCBI nucleotide database. MEGA X [24] was used to edit and align the nucleotide and amino acid sequences and to calculate the pairwise identities of the genomes and all ORFs that were predicted using the online tool ORF Finder (NCBI, http://www.ncbi.nlm.nih.gov/gorf/gorf.html, accessed on 9 February 2026). Maximum likelihood phylogenetic trees were inferred using IQ-TREE 2 [25], with ModelTest used to determine the best-fitting substitution model for each dataset, and were run with 1000 bootstrap replicates.

### 2.6. Secondary Structure Alignment

The secondary structure elements were annotated using the ESPript online tool (http://espript.ibcp.fr) [26] by aligning the receptor-binding domain (RBD) regions of SARS-CoV, SARS-CoV-2, related strains and Sarbecovirus-related bat strains.

## 3. Results

### 3.1. Molecular Screening of Coronavirus

None of the 37 bat fecal samples analyzed tested positive for SARS-CoV-2. Using the real-time RT-PCR for Sarbecovirus, a Beta-CoV subgenus, 14 *R. hipposideros* fecal samples tested positive (14/37, 37.8%), albeit with high cycle threshold (Ct) values (>33) (Table 1). These samples were collected from three roosts (PIN, VIT1 and VIT2) located in the provinces of Pordenone and Treviso (Supplementary Table S1).

All bat samples were further screened using a Pan-CoV nested RT-PCR to amplify a short genomic sequence within the RdRp region. The test identified 3 additional positive samples from the Sarbecovirus-negative group (3/37, 8.1%), classified as Alpha-CoV, while 2 samples were confirmed as positive within the group of 14 Sarbecovirus-positive samples (Table 1). These 3 samples were all collected from the same roost (OS) in the province of Udine. In total, 4 of the 5 roosts were positive for the presence of CoVs (Supplementary Table S1).

Blastn analysis of the three RdRp sequences obtained from the Sarbecovirus-negative samples revealed similarities to Alpha-CoV strains (designated Bat/OS02MYP/UD2020, Bat/OS06MSP/UD2020, and Bat/OS07MSP/UD2020). These three sequences shared 100% nucleotide identity (nt.id.) and were all collected from the same roost in June 2020. Blastn results showed high nucleotide identities to four European strains: three from Spain: PQ611140 (MsAlphaCoV_Gordo), PQ738186.1 (MsAlphaCoV-Spain2), and HQ184061.1 (H.sav/L/Spain/2007), detected in *M. schreibersii* and *Hypsugo savii (H. savii),* and one from a *Miniopterus spp.* collected in 2023 in France (PV125529). The nucleotide identity with these strains ranged from 96.2% to 97.1%.

Two RdRp sequences obtained from Sarbecovirus-positive samples (Bat/PIN21RIP/PN2020, Bat/VIT11RIP/TV2020) were identical (100% nt.id.) despite originating from two different colonies. Blastn analysis revealed that these RdRp sequences displayed the highest nucleotide identity (99.7% nt.id.) with a bat CoV sequence previously identified in Slovenia in 2008 from *R. hipposideros* (SLO1A0082/2008/SVN, GQ404797), a SARS-CoV-related coronavirus.

### 3.2. Genome Reconstruction and Detection of Multiple Coronavirus Lineages

Eight coronavirus-positive samples were selected for targeted Illumina sequencing using the Pan-CoV kit, including 3 Alpha-CoV-positive and 5 Sarbecovirus-positive samples (the latter characterized by high Ct values). Near-complete viral genomes could be reconstructed from only two samples, namely Bat/OS06MSP/UD2020 and Bat/PIN21RIP/PN2020.

The sequencing runs generated 943,488 and 1,551,996 paired-end reads, respectively. Between 8338 and 72,407 reads mapped to coronavirus reference genomes, resulting in mean genome coverages ranging from 32.1× to 68.1×. In the remaining six samples, despite a comparable sequencing output, fewer than 100 reads mapped to coronavirus references, which precluded meaningful genome reconstruction.

All genomes displayed the canonical CoV genome organization (5′UTR–ORF1ab–S–E–M–N–3′UTR).

Three nearly complete Alpha-CoV genomes were recovered from sample Bat/OS06MSP/UD2020. One strain (Bat/OS06MSP/UD2020A) was represented by a 20,938-nt partial ORF1ab sequence, whereas strains Bat/OS06MSP/UD2020B and Bat/OS06MSP/UD2020C yielded near-complete genomes of approximately 30 kb, lacking only 706 and 1514 nucleotides, respectively. The RdRp fragment obtained by Pan-CoV nested RT-PCR was identical to the corresponding region of strain Bat/OS06MSP/UD2020A, likely reflecting preferential amplification by the degenerate RT-PCR primers or differential viral abundance in the sample.

Pairwise comparison revealed substantial divergence among the three co-circulating strains, with nucleotide identities ranging from 72.3% to 78.1%.

Phylogenetic analysis based on complete genome sequences revealed the separation of the 3 Alpha-CoV sequences in 3 clusters (Supplementary Figure S1).

Bat/OS06MSP/UD2020A clustered with a Spanish strain (PQ611140) from 2022 reported in *M. schreibersii* (98.7% nt.id.), whereas Bat/OS06MSP/UD2020B clustered with a Chinese strain from 2020 (OQ175102) from *Myotis adversus* (*M. adversus*) (89.6% nt.id.)*,* and Bat/OS06MSP/UD2020C clustered with two Chinese strains: one reported in *Miniopterus fuliginosus* (*M. fuliginosus*) in 2011 (KJ473795) and the reference sequence for *Alphacoronavirus pusilli* from *Miniopterus magnater* (*M. magnater*) (NC_010437), with 90.6% and 90.2% nt.id., respectively (Supplementary Figure S1).

A near-complete Beta-CoV genome was recovered from sample Bat/PIN21RIP/PN2020. The reconstructed genome was approximately 25 kb long, lacking 5059 nucleotides. The phylogenetic analysis showed its clustering with a Spanish strain reported in *R. hipposideros* in 2022, sharing 94.0% nucleotide identity (PQ611137) (Supplementary Figure S2).

### 3.3. Phylogenetic Analysis

**Figure 2:**
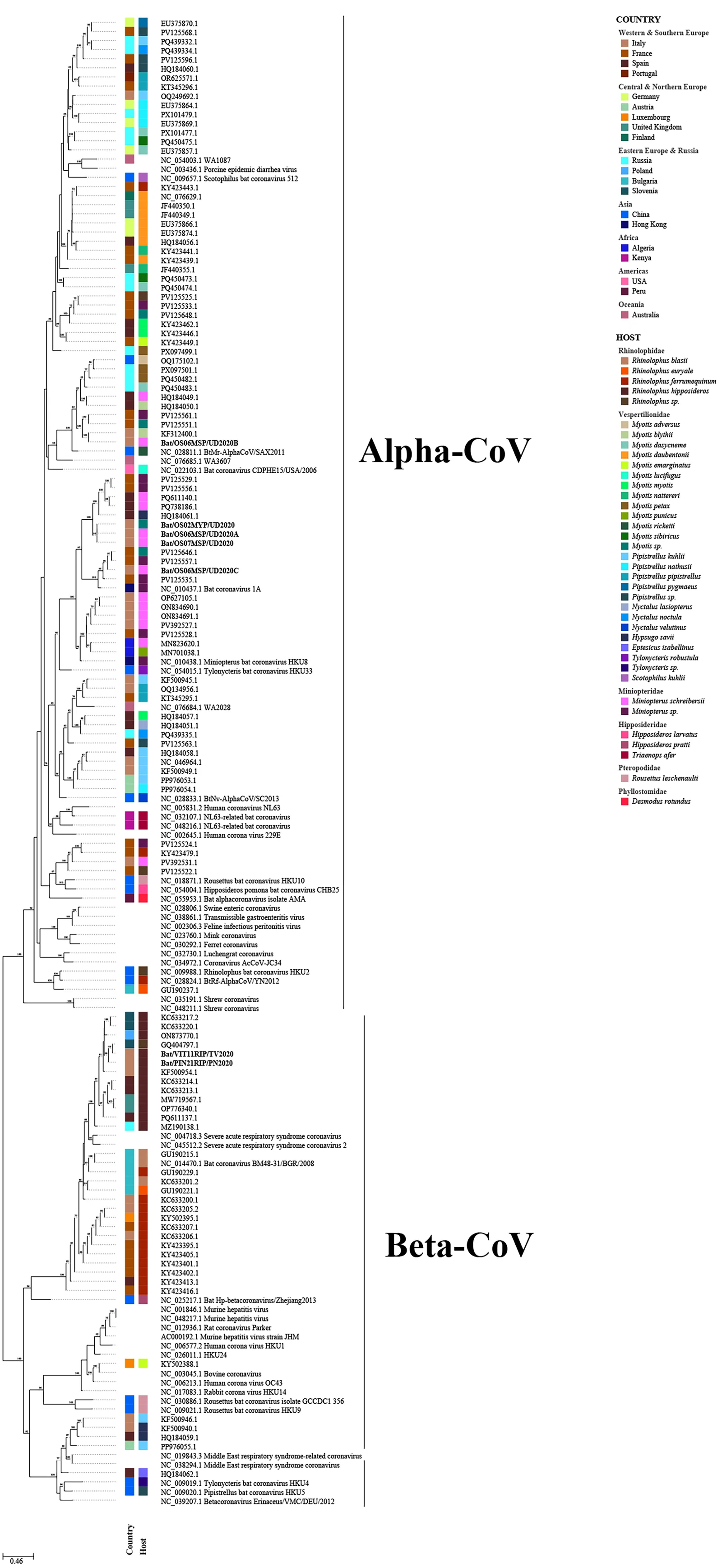
Phylogenetic tree built using a short genomic sequence within the RdRp region (Dataset C). The maximum likelihood tree was built using the GTR + F + I + G4 model based on 1000 bootstrap replications, with bootstrap values > 70 indicated at their respective nodes.

Phylogenetic analysis based on partial RdRp sequences revealed distinct clustering patterns among the three Alpha-CoV sequences from this study (Figure 2). Bat/OS02MYP/UD2020, Bat/OS06MSP/UD2020A, and Bat/OS07MSP/UD2020 sequences clustered together with five European sequences: two French strains from *Miniopterus* spp. (PV125529, PV125556) and three Spanish strains from *M. schreibersii* and *H. savii* (PQ611140, PQ738186, HQ184061), sharing nucleotide identities of between 96.4% and 97.3%.

The Bat/OS06MSP/UD2020B sequence clustered within a larger group of eight bat-derived Alpha-CoVs, which subdivided into two distinct sub-clusters. Sub-cluster 1 included Bat/OS06MSP/UD2020B, closely related to one Italian strain from *M. blythii* (KF312400) and two French strains from *Miniopterus* spp. and *Myotis* spp. (PV125561, PV125551), with nucleotide identities ranging from 96.7% to 98.9%. Sub-cluster 2 comprised three Russian strains from *Myotis petax (M. petax)* and *Myotis dasycneme* (*M. dasycneme*) (PX097501, PQ450482, PQ450483) and two Spanish strains from *M. schreibersii* and *M. blythii* (HQ184049, HQ184050), showing lower identity scores (91.0–92.0%).

In contrast, Bat/OS06MSP/UD2020C clustered separately with three French sequences from *Myotis* spp. and *Miniopterus* spp. (PV125646, PV125557, PV125535), displaying 95.6–98.9% nucleotide identities. This cluster also included the reference sequence for *Alphacoronavirus pusilli*, originally described in *M. magnater* from China (NC_010437), which shared 89.5% identity with Bat/OS06MSP/UD2020C.

The two Sarbecovirus sequences (Bat/PIN21RIP/PN2020 and Bat/VIT11RIP/TV2020) clustered with Beta-CoV sequences predominantly from Central and Southern Europe, including strains from Slovenia (KC633217, KC633220, GQ404797), Poland (ON873770), and Italy (KF500954), all isolated from *R. hipposideros* and *Rhinolophus* spp., with high similarity scores ranging from 95.3% to 99.7% (Figure 2).

Overall, phylogenetic analyses consistently demonstrated that all coronavirus strains identified in this study clustered within the well-characterized European coronavirus diversity, with no evidence of recent introduction of highly divergent or novel lineages.

### 3.4. Receptor-Binding Domain Analysis

Since the Alpha-CoV sequences lacked complete spike gene and RBD sequences, a comparative sequence analysis of the RBD, including the annotation of secondary structure elements, was carried out exclusively on the RBD derived from the Sarbecovirus sequence Bat/PIN21RIP/PN2020.

At the nucleotide level, the RBD of Bat/PIN21RIP/PN2020 displayed high identity with RhBetaCoV_Murcia2022 (97.0%) but lower identities with SARS-CoV (68.1%, NC_004718.3) and SARS-CoV-2 (65%, NC_045512.2) reference sequences. Corresponding amino acid identities were 96.2%, 69.3%, and 66.9%, respectively.

Table 2 summarizes the critical amino acid residues involved in human angiotensin-converting enzyme 2 (hACE2) receptor binding. Notably, Bat/PIN21RIP/PN2020 shares minimal similarity with SARS-CoV (0/5 conserved residues) but retains four of the seven key ACE2-binding residues found in SARS-CoV-2 (positions 455, 486, 499, and 505). In contrast, all seven residues are identical to those of RhBetaCoV_Murcia2022, a bat Sarbecovirus reported to bind hACE2 in vitro.

Three positions differ between Bat/PIN21RIP/PN2020 and SARS-CoV-2: position 493 (Q → A), position 494 (S → D), and position 501 (N → S). The Q493A substitution replaces a polar uncharged residue with a small hydrophobic one, potentially eliminating hydrogen bonding capacity. The S494D change introduces a negatively charged aspartic acid in place of a polar uncharged serine. The N501S substitution exchanges an asparagine with a smaller serine residue, which may create a structural gap at the binding interface.

Alignment of the annotated secondary structure elements indicated that the main difference between Bat/PIN21RIP/PN2020 and the SARS-CoV-2 RBD template is a 4-amino acid deletion in the loop region between β2 and α4 secondary structures. This deletion shortens the loop compared to SARS-CoV-2. Notably, a comparable shortening of this loop is also observed in other bat Sarbecoviruses capable of using ACE2 (Supplementary Figure S3), indicating that this feature is not exclusive to our strain.

## 4. Discussion

Bats serve as natural reservoirs for numerous zoonotic pathogens that threaten both human and animal health. The COVID-19 pandemic, caused by the Sarbecovirus SARS-CoV-2, has intensified the scientific focus on the diversity and geographic distribution of CoVs within chiropteran populations. Surveillance of wildlife close to anthropogenic environments remains a critical pillar of pandemic preparedness and of “One Health” strategies.

In this study, a bat population in Northern Italy was investigated for the presence of CoVs. Sampling was conducted in June 2020, immediately after the national COVID-19 lockdown.

The Pan-CoV screening, performed by Sarbecovirus real-time RT-PCR and nested RT-PCR targeting the RdRp region, identified an overall CoV positivity of 45.9% (17/37). While global meta-analyses often report an average bat CoV prevalence of approximately 10.0–20.0% (e.g., *Myotis*, *Rhinolophus*, and *Pipistrellus* complexes), European data show considerable regional variation [27,28]. Lower prevalences have been in other European studies, for example 9.8% in Northern Germany [6], 5.5% in Spain [29] and 0% in Belgium [30]. Notably, higher prevalences have been reported in the United Kingdom (23.2%) [28] and the Netherlands (16.9%) [31]. These geographic differences probably reflect variation in sampling season, colony density, bat species composition and detection methodology. Recent Italian studies illustrate this regional heterogeneity, with detection rates ranging from 2.7% to 12.6% and with both Alpha-CoVs and Beta-CoVs frequently identified [7,8,9,11].

The elevated proportion of positive samples reported here (45.9%) appears to be primarily driven by the high frequency of Sarbecovirus detection. Several factors likely contribute to this finding: the enhanced sensitivity of real-time RT-PCR compared to conventional Pan-CoV screening approaches used in many previous studies; the high prevalence of Sarbecovirus strains in the surveyed bat colonies, which may reflect local ecological or epidemiological conditions; and the targeted sampling of colonies with high bat density, potentially increasing viral circulation opportunities. In contrast, the proportion of Alpha-CoV positive samples (8.1%) remains comparable to published European data, supporting the hypothesis that the elevated overall positivity is largely attributable to methodological advantages in Sarbecovirus detection rather than extraordinary local viral diversity.

The presence of Sarbecovirus strains in *R. hipposideros* confirms the established understanding that horseshoe bats act as natural reservoirs for this subgenus across Europe, with reported prevalences of 38.8% in Slovenia [32], 43.5–54.8% in the United Kingdom [33], 62.5% in Russia [34] and 31.0% in Poland [35]; infection rates vary considerably by site and sampling period.

Sequence analysis of the RdRp region revealed the presence of viruses belonging to two genera, Alphacoronavirus and Betacoronavirus. The Alpha-CoVs detected here in *M. schreibersii* and *Myotis* spp. were related to three Spanish strains and one French strain reported between 2007 and 2023. These related strains had been reported in *M. schreibersii, Miniopterus* sp. and *H. savii* bats. Alpha-CoVs in *M. schreibersii* have been extensively documented across Europe and the Mediterranean, with reports from Bulgaria [36], France and Spain [37], Portugal [38], and Lebanon [39]. In Italy, their presence has been confirmed by more recent surveillance in Tuscany and Latium [12] and in Sicily, which identified novel Alpha-CoV strains closely related to those circulating in the wider Mediterranean basin [11]. The high diversity of Alpha-CoV strains identified in European *M. schreibersii* probably reflects the highly gregarious nature of this species and its capacity for long-distance regional migration. In *Myotis* spp., Alpha-CoVs have been described in Germany [6,31], Denmark [27], Portugal [38], the Netherlands [31], Spain [29,37], France [37] and Bulgaria [36], as well as in Northern Italy [8,9]. In contrast, the *Myotis* genus comprises ecologically heterogeneous, largely sedentary or regionally moving species, and Alpha-CoVs detected in European *Myotis* bats typically cluster by host species across countries rather than by geographic origin, a pattern consistent with virus–host co-divergence and host restriction [27].

The Beta-CoV strain identified in this study was detected in two *R. hipposideros* fecal samples. Phylogenetic analysis indicates a high degree of sequence identity with a SARS-CoV-related coronavirus previously identified in a Slovenian *R. hipposideros* in 2008 [32]. In Italy, the circulation of Sarbecoviruses in *R. hipposideros* has remained relatively poorly documented. Beyond the findings of Mira et al. (2025) [11] in Sicily, these strains were previously reported in Northern Italy during the 2011–2012 surveillance period [7]. SARS-CoV-related Beta-CoVs have also been reported in Sardinia, where a single strain was detected in *R. ferrumequinum*, *Plecotus auritus* and *Tadarida teniotis* [15]. To characterize the detected CoV strains, a targeted Pan-CoV Illumina approach was applied to enrich for *Coronaviridae*-specific sequences. For the samples positive for Sarbecovirus by real-time RT-PCR, selection for next-generation sequencing (NGS) was based on viral load; specifically, samples with the lowest Ct values (representing the highest viral loads) were prioritized.

For the Alpha-CoV-positive samples, selection could not be guided by quantitative data due to the lack of a specific Alpha-CoV real-time assay. Instead, all three samples positive by nested Pan-CoV PCR were processed for sequencing.

Given the relatively high Ct values for the Sarbecovirus strains and the presumably low viral titers in the Alpha-CoV samples, only two samples produced sufficient read depth to construct long contigs and near-complete genomes. Attempts were made to resolve remaining genomic gaps using de novo-designed gap-filling PCR primers; however, these efforts did not yield successful amplifications. This suggests that the integrity or the concentration of viral RNA in the fecal swabs was at the limit of detection of current protocols.

The NGS and subsequent bioinformatic analysis identified three distinct Alpha-CoV strains within a single fecal sample (*M. schreibersii*, sample Bat/OS06MSP/UD2020). Because fecal material was collected beneath roosting clusters, it cannot be determined whether the three Alpha-CoV strains originated from a single co-infected bat or from fecal material contributed by more than one individual.

One partial genome, designated Bat/OS06MSP/UD2020A, showed 100% sequence identity in the RdRp region to the sequences initially obtained via Pan-CoV nested RT-PCR and Sanger sequencing. This result supports the accuracy of our initial screening; however, the detection of two additional strains in the same sample highlights the inherent limitations of Sanger sequencing.

Whereas Sanger sequencing typically captures only the dominant “consensus” sequence in a multi-strain infection, NGS provides the depth and resolution required to detect minor variants and to characterize the full viral diversity present. These results underscore the value of high-throughput sequencing for resolving the true heterogeneity of coronavirus populations in wildlife reservoirs.

At the complete genome level, Bat/OS06MSP/UD2020A was most closely related to a Spanish strain from 2022 from *M. schreibersii*, whereas Bat/OS06MSP/UD2020B and Bat/OS06MSP/UD2020C showed approximately 90% nt.id. with strains previously documented in China (from *M. adversus*, *M. fuliginosus* and *M. magnater*), suggesting a distant common ancestor rather than recent transmission.

At the RdRp level, Bat/OS06MSP/UD2020B and Bat/OS06MSP/UD2020C were phylogenetically related to European Alpha-CoV strains. In particular, both strains were related to Alpha-CoV strains found in France in *Myotis* spp. and *Miniopterus* spp. Furthermore, Bat/OS06MSP/UD2020B was also related to an Italian strain found in Northern Italy in 2012 from a *M. blythii* bat, and to two French strains found in *Miniopterus* spp. and *Myotis* spp.

These findings confirm that, despite the importance of full-genome sequencing, the RdRp region, the genomic region most commonly amplified for screening purposes, remains a robust and reliable molecular marker for identifying evolutionary lineages and tracking viral distribution.

Our results document the simultaneous presence of three distinct Alpha-CoV strains within feces collected from animals living in the same roost, indicating the local co-circulation of genetically distinct Alpha-CoV lineages. Given the relatively high frequency of CoV co-infections in bats, the assembled genomes were carefully inspected to exclude the presence of chimeric sequences. This evaluation was based on the consistency of read coverage and the absence of significant heterogeneity within each assembly. In addition, blast comparisons among the reconstructed genomes did not reveal identical sequences, further supporting that the detected genomes were genetically distinct rather than artifacts of misassembly. The co-circulation and co-infection of multiple CoV lineages have previously been documented at different ecological scales, including within individual bats and across sympatric bat populations, including in *Miniopterus* spp. [40,41]. The simultaneous presence of multiple CoV species or strains within a single host significantly increases the probability of genetic recombination. Strains belonging to the Alpha-CoV or Beta-CoV genera, or to both, have been described in the same animal, a situation that may lead to homologous recombination between related CoV strains or to heterologous recombination between CoVs from different families.

As reviewed by Wong et al. (2025) [41], recombination events, particularly within the RBD region, can alter the cell types or host species that a virus is able to infect (e.g., *BatCoV HKU10*), increase the binding affinity of the virus for host receptors (e.g., the relationship between *MERS-CoV* and *Ty-BatCoV HKU4*), or modify susceptibility to antibody neutralization, as observed for SARS-CoV-2 variants, which can reduce the protection conferred by post-vaccination sera.

At the complete genome level, the Beta-CoV sequence reported here (Bat/PIN21RIP/PN2020) was related to a SARS-CoV-related strain found in Spain in 2022 in *R. hipposideros*. The RdRp and complete genome analyses confirm the relationship with European strains (Poland and Slovenia) and with an Italian strain found in Northern Italy in an *R. hipposideros* bat in 2012. The strains reported here were found in two fecal samples collected from two different roosts almost 50 km apart. This highlights the geographical persistence of SARS-CoV-related strains across the Alpine and Mediterranean corridors within this bat species. Since *R. hipposideros* is a sedentary species, the SARS-CoV-related coronavirus strain identified is likely endemic to specific regions. However, given the close phylogenetic relationship with other European strains, we cannot exclude an introduction by a migratory “visitor” species.

While early studies suggested that host species specificity could reliably predict the geographic distribution of CoVs, recent evidence indicates a far more complex ecological scenario. Current literature suggests that viral evolution is driven by dynamic co-evolutionary processes between the virus and its host, which are further modulated by the ethological traits and social structures of bat colonies [42]. In our study, owing to the limited sample size and to the detection of CoVs in only two bat species, we observed the circulation of specific viral genera restricted to single host species. This contrasts with the findings of Mira et al. (2025) [11], who reported a broader cross-species distribution. Consequently, our data underscore the difficulty of confirming a single dominant hypothesis regarding host restriction versus viral plasticity in the absence of broader longitudinal data. A further limitation concerns the non-invasive sampling strategy: because feces were collected from sheets placed beneath roosting clusters, samples cannot be assigned to individual bats. Host species assignment was based on the known species composition of the monitored colonies and was therefore less certain in mixed-species roosts. Prevalence values consequently refer to the proportion of positive fecal samples and not to the proportion of infected animals, and the two may differ if multiple samples originated from the same bat. In addition, the limited number of samples and the single sampling season mean that the coronavirus diversity reported here should be regarded as a partial picture of that circulating in the study area.

The comparative sequence analysis of the RBD of the SARS-CoV-related strain showed that Bat/PIN21RIP/PN2020 shared 4 amino acid residues with the SARS-CoV-2 RBD and that all the residues examined were identical to those of RhBetaCoV_Murcia2022. Soriano-Tordera et al. (2025) [43] showed that the latter strain was able to use the hACE2 receptor, although with weaker binding than SARS-CoV-2. It must be stressed, however, that identity at these positions is not in itself evidence of receptor usage: hACE2 binding of RhBetaCoV_Murcia2022 was established experimentally, whereas for Bat/PIN21RIP/PN2020 only sequence-level comparisons are available. Whether this strain binds hACE2 therefore remains an open question that can only be settled by structural modeling and, ultimately, by pseudovirus or receptor-binding assays, which were beyond the scope of the present surveillance study. In recent years, several SARS-CoV-related lineages have been found to bind hACE2 despite divergence within the RBD, including lineages from bats in Spain, Russia, Kenya and China [34,43,44,45,46]. While Sarbecovirus lineages share a common evolutionary architecture, their RBDs show some divergence. As stated by Soriano-Tordera et al. (2025) [43], the mere presence of these viral lineages does not imply an immediate capacity for human infection, nor does it indicate that the threshold for a spillover event has been met. Our RBD comparison should accordingly be read as a descriptive, hypothesis-generating observation that may help prioritize strains for future functional characterization, and not as evidence of zoonotic potential.

To date, few studies have reported SARS-CoV-2-related strains in bats; the most closely related strain, RaTG13, was described in *Rhinolophus affinis* from China and shares 96.2% nucleotide identity with SARS-CoV-2 [47], while a further related strain, RmYN02, was identified in *Rhinolophus malayanus* [48]. The absence of SARS-CoV-2 in the present study adds to the growing evidence that spillback events to wild bats may be rare or absent in natural settings, even in European populations exposed to high human infection rates during the pandemic.

## 5. Conclusions

In conclusion, our findings emphasize the role of bats as a global reservoir of a diverse array of coronaviruses. Future efforts to characterize viral diversity across different hosts and regions, together with ecological monitoring of the interactions between bats and humans, will be essential to mitigate the risk of future pandemics.

## Figures and Tables

**Table 1:** Results of molecular tests.

Bat Species	N Samples	Real Time RT-PCR SARS-CoV-2	Real Time RT-PCR Sarbecovirus	Nested RT-PCR PanCoV	Positivity Rate
*Miniopterus schreibersii*	4	0	0	2	50.0%
*Myotis daubentonii*	2	0	0	0	0
*Myotis emarginatus*	7	0	0	0	0
*Myotis* spp.	4	0	0	1	25.0%
*Rhinolophus hipposideros*	20	0	14	2 *	70.0%
**Total**	**37**	**0**	**14/37 (37.8%)**	**5/37 (13.5%)**	**17/37 (45.9%)**

* positive samples confirmed by both Real Time RT-PCR and Nested RT-PCR PanCoV.

**Table 2:** Variations in key amino acid residues involved in binding to the human ACE2 receptor. Differences among SARS-CoV, SARS-CoV-2, and related bat coronavirus strains are reported.

Amino Acids Positions *									
								CoVs **	
	T487			Y484	N479	L472	Y442	SARS-CoV	
Y505	N501	P499	S494		Q493	F486	L455	SARS-CoV-2	
Y	S	P	D	T	A	F	L	**Bat/PIN21RIP/PN2020**	
F	S	Q	S	T	A	L	R	**BM48-31/BGR/2008**	
F	S	Q	S	T	K	L	L	**BatCoV/BB9904/BGR/2008**	
Y	S	T	D	Y	K	L	Y	**Civet SZ3**	**SARS-CoV-related**
Y	T	P	R	H	E	L	L	**Pangolin CoV**	**SARS-CoV-2-related**
H	D	P	R	Y	Y	L	L	**RaTG13**	
Y	S	P	D	T	A	F	L	**RhBetaCoV_Murcia2022**	ACE+ ***
Y	V	P	S	T	K	L	L	**BtKY72**	
Y	S	S	D	T	Q	H	L	**Khosta-2**	
Y	N	I	D	Y	N	F	S	**WIV1**	
Y	N	I	D	Y	N	F	S	**WIV16**	
H	A	T	P	F	R	P	W	**RsSHC014**	
Y	N	T	D	F	N	F	S	**Rs7327**	
Y	V	P	T	N	S	G	S	**HKU3-1**	**ACE−**
Y	V	P	T	N	S	-	S	**Rf4092**	
Y	V	P	T	Y	S	-	S	**Rs4237**	
Y	V	P	T	N	S	G	S	**Rs4247**	

* A—Alanine; R—Arginine; N—Asparagine; D—Aspartic acid; F—Phenylalanine; G—Glycine; Q—Glutamine; E—Glutamic acid; H—Histidine; I—Isoleucine; L—Leucine; K—Lysine; P—Proline; S—Serine; T—Threonine; Y—Tyrosine; W—Tryptophan; V—Valine. ** Accession numbers: SARS-CoV, YP_009825051; SARS-CoV-2, YP_009724390; BM48-31/BGR/2008, YP_003858584; BatCoV/BB9904/BGR/2008, ALJ94036; Civet SZ3, AY304486; Pangolin CoV, QIA48632; RaTG13, QHR63300; RhBetaCoV_Murcia2022, PQ611137; BtKY72, APO40579; Khosta-2, QVN46559; WIV1, AGZ48831; WIV16, ALK02457; RsSHC014, AGZ48806; Rs7327, ATO98218; HKU3-1, AAY88866; Rf4092, ATO98145; Rs4237, ATO98169; Rs4247, ATO98181. *** ACE+ and ACE− denote, respectively, strains whose spike protein has been shown to bind the human ACE2 receptor in vitro, and strains for which such binding was not detected.

## Data Availability

All sequences produced in this study were submitted to GenBank database. Accession numbers: PZ668198-PZ668201, PZ668202-PZ668204.

## Supplementary Materials

The following supporting information can be downloaded at: https://www.mdpi.com/article/10.3390/ani16203319/s1, Figure S1: Phylogenetic tree built using Alpha-CoV complete genomes (Dataset A); Figure S2: Phylogenetic tree built using Beta-CoV complete genomes (Dataset B); Figure S3: Alignment of amino acid sequences and secondary structure of Sarbecovirus RBD protein sequences. Table S1: Overview of bat roosts sampled in this study.

## Abbreviations

The following abbreviations are used in this manuscript:

ACE2	Angiotensin-converting enzyme 2
Alpha-CoV	Alphacoronavirus
Beta-CoV	Betacoronavirus
CoV	Coronavirus
Ct	Cycle threshold
*H. savii*	*Hypsugo savii*
hACE2	Human angiotensin-converting enzyme 2
*M. adversus*	*Myotis adversus*
*M. blythii*	*Myotis blythii*
*M. dasycneme*	*Myotis dasycneme*
*M. daubentonii*	*Myotis daubentonii*
*M. emarginatus*	*Myotis emarginatus*
*M. fuliginosus*	*Miniopterus fuliginosus*
*M. magnater*	*Miniopterus magnater*
*M. myotis*	*Myotis myotis*
*M. petax*	*Myotis petax*
*M. schreibersii*	*Miniopterus schreibersii*
MERS-CoV	Middle East respiratory syndrome coronavirus
NGS	Next-generation sequencing
nt.id.	nucleotide identity
Pan-CoV	Pan-coronavirus
PPE	Personal protective equipment
*R. ferrumequinum*	*Rhinolophus ferrumequinum*
*R. hipposideros*	*Rhinolophus hipposideros*
RBD	Receptor-binding domain
RdRp	RNA-dependent RNA polymerase
RT-PCR	Reverse transcription polymerase chain reaction
SARS-CoV	Severe acute respiratory syndrome coronavirus
SARS-CoV-2	Severe acute respiratory syndrome coronavirus 2
spp.	species
