# Supplementary material for: Coronavirus Diversity in an Italian Bat Population Sampled During the Pandemic in 2020
Source: Animals (Basel). 2026 Oct 21;16(20):3319. doi: 10.3390/ani16203319 (PMC13700689; doi:10.3390/ani16203319)
Supplement: Supplementary file 1 [file animals-16-03319-s001.zip › SupplFigS3.pdf]

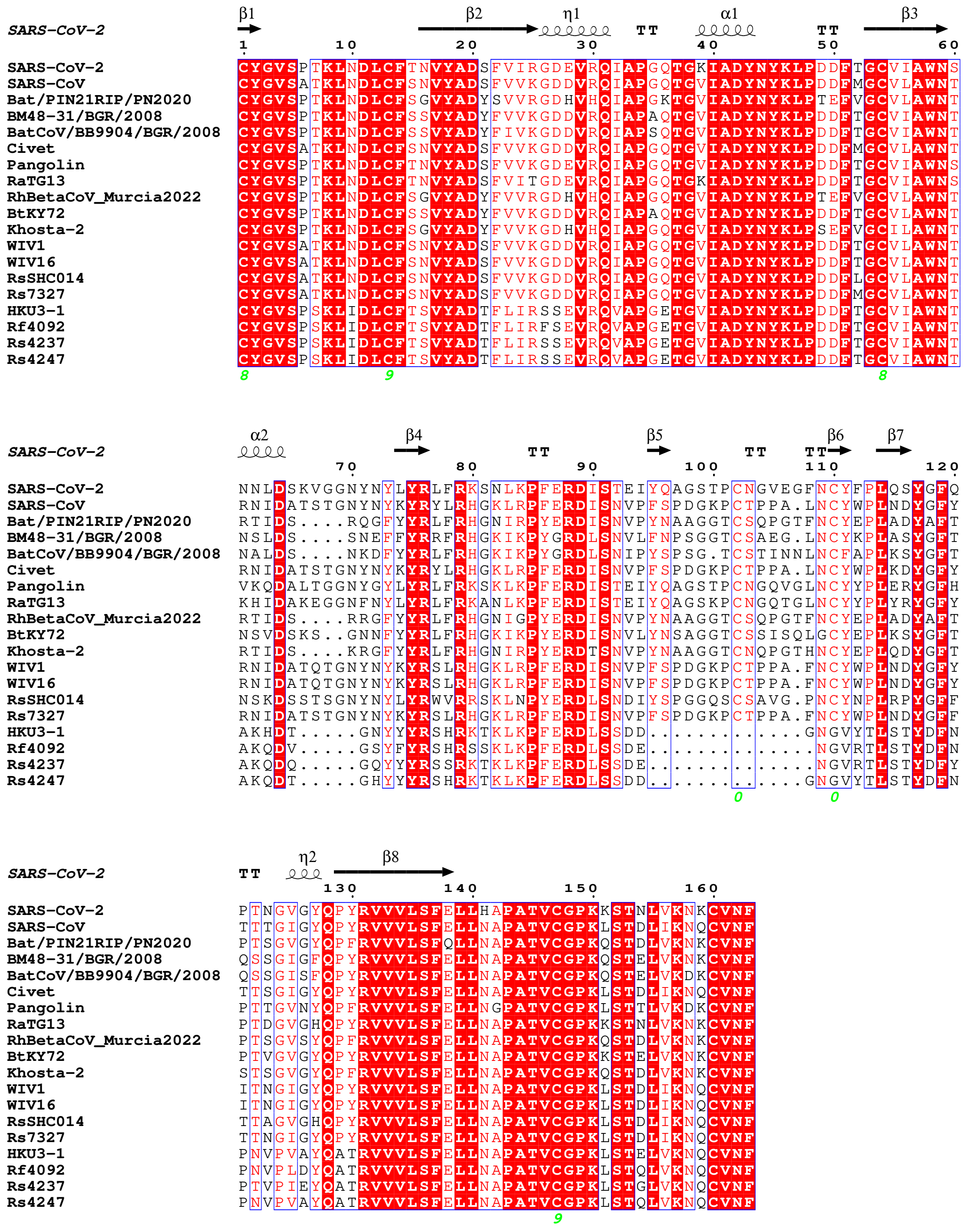

SARS-CoV-2
SARS-CoV-2 CYGVSPTKLNDLCFTNVYADSFVIRGDEVRQIAPGQTGKIADYNYKLPDDFTGCVIAWNS
SARS-CoV CYGVSATKLNDLCFSNVYADSFVVKGDDVRQIAPGQTGVIADYNYKLPDDFMGCVLAWNT
Bat/PIN21RIP/PN2020 CYGVSPTKLNDLCFSGVYADYSVVRGDHVHQIAPGKTGVIADYNYKLPTEFVGCVLAWNT
BM48-31/BGR/2008 CYGVSPTKLNDLCFSSVYADYFVVKGDDVRQIAPAQTGVIADYNYKLPDDFTGCVIAWNT
BatCoV/BB9904/BGR/2008 CYGVSPTKLNDLCFSSVYADYFIVKGDDVRQIAPSQTGVIADYNYKLPDDFTGCVIAWNT
Civet CYGVSATKLNDLCFSNVYADSFVVKGDDVRQIAPGQTGVIADYNYKLPDDFMGCVLAWNT
Pangolin CYGVSPTKLNDLCFTNVYADSFVVKGDEVRQIAPGQTGVIADYNYKLPDDFTGCVIAWNS
RaTG13 CYGVSPTKLNDLCFTNVYADSFVITGDEVRQIAPGQTGKIADYNYKLPDDFTGCVIAWNS
RhBetaCoV_Murcia2022 CYGVSPTKLNDLCFSGVYADYFVVRGDHVHQIAPGQTGVIADYNYKLPTEFVGCVLAWNT
BtKY72 CYGVSPTKLNDLCFSSVYADYFVVKGDDVRQIAPAQTGVIADYNYKLPDDFTGCVLAWNT
Khosta-2 CYGVSPTKLNDLCFSGVYADYFVVKGDHVHQIAPGQTGVIADYNYKLPSEFVGCILAWNT
WIV1 CYGVSATKLNDLCFSNVYADSFVVKGDDVRQIAPGQTGVIADYNYKLPDDFTGCVLAWNT
WIV16 CYGVSATKLNDLCFSNVYADSFVVKGDDVRQIAPGQTGVIADYNYKLPDDFTGCVLAWNT
RsSHC014 CYGVSATKLNDLCFSNVYADSFVVKGDDVRQIAPGQTGVIADYNYKLPDDFLGCVLAWNT
Rs7327 CYGVSATKLNDLCFSNVYADSFVVKGDDVRQIAPGQTGVIADYNYKLPDDFMGCVLAWNT
HKU3-1 CYGVSPSKLIDLCFTSVYADTFLIRSSEVRQVAPGETGVIADYNYKLPDDFTGCVIAWNT
Rf4092 CYGVSPSKLIDLCFTSVYADTFLIRFSEVRQVAPGETGVIADYNYKLPDDFTGCVIAWNT
Rs4237 CYGVSPSKLIDLCFTSVYADTFLIRSSEVRQVAPGETGVIADYNYKLPDDFTGCVIAWNT
Rs4247 CYGVSPSKLIDLCFTSVYADTFLIRSSEVRQVAPGETGVIADYNYKLPDDFTGCVIAWNT
SARS-CoV-2
SARS-CoV-2 NNLDSKVGGNYNYLYRLFRKSNLKPFERDISTEIYQAGSTPCNGVEGFNCYFPLQSYGFQ
SARS-CoV RNIDATSTGNYNYKYRYLRHGKLRPFERDISNVPFSPDGKPCTPPA.LNCYWPLNDYGFY
Bat/PIN21RIP/PN2020 RTIDS....RQGFYYRLFRHGNIRPYERDISNVPYNAAGGTCSQPGTFNCYEPLADYAFT
BM48-31/BGR/2008 NSLDS....SNEFFYRRFRHGKIKPYGRDLSNVLFNPSGGTCSAEG.LNCYKPLASYGFT
BatCoV/BB9904/BGR/2008 NALDS....NKDFYYRLFRHGKIKPYGRDLSNIPYSPSG.TCSTINNLNCFAPLKSYGFT
Civet RNIDATSTGNYNYKYRYLRHGKLRPFERDISNVPFSPDGKPCTPPA.LNCYWPLKDYGFY
Pangolin VKQDALTGGNYGYLYRLFRKSKLKPFERDISTEIYQAGSTPCNGQVGLNCYYPLERYGFH
RaTG13 KHIDAKEGGNFNYLYRLFRKANLKPFERDISTEIYQAGSKPCNGQTGLNCYYPLYRYGFY
RhBetaCoV_Murcia2022 RTIDS....RRGFYYRLFRHGNIGPYERDISNVPYNAAGGTCSQPGTFNCYEPLADYAFT
BtKY72 NSVDSKS..GNNFYYRLFRHGKIKPYERDISNVLYNSAGGTCSSISQLGCYEPLKSYGFT
Khosta-2 RTIDS....KRGFYYRLFRHGNIRPYERDTSNVPYNAAGGTCNQPGTHNCYEPLQDYGFT
WIV1 RNIDATQTGNYNYKYRSLRHGKLRPFERDISNVPFSPDGKPCTPPA.FNCYWPLNDYGFY
WIV16 RNIDATQTGNYNYKYRSLRHGKLRPFERDISNVPFSPDGKPCTPPA.FNCYWPLNDYGFY
RsSHC014 NSKDSSTSGNYNYLYRWVRRSKLNPYERDLSNDIYSPGGQSCSAVG.PNCYNPLRPYGFF
Rs7327 RNIDATSTGNYNYKYRSLRHGKLRPFERDISNVPFSPDGKPCTPPA.FNCYWPLNDYGFF
HKU3-1 AKHDT.....GNYYYRSHRKTKLKPFERDLSSDD.............GNGVYTLSTYDFN
Rf4092 AKQDV.....GSYFYRSHRSSKLKPFERDLSSDE..............NGVRTLSTYDFN
Rs4237 AKQDQ.....GQYYYRSSRKTKLKPFERDLSSDE..............NGVRTLSTYDFY
Rs4247 AKQDT.....GHYYYRSHRKTKLKPFERDLSSDD.............GNGVYTLSTYDFN
SARS-CoV-2
SARS-CoV-2 PTNGVGYQPYRVVVLSFELLHAPATVCGPKKSTNLVKNKCVNF
SARS-CoV TTTGIGYQPYRVVVLSFELLNAPATVCGPKLSTDLIKNQCVNF
Bat/PIN21RIP/PN2020 PTSGVGYQPFRVVVLSFQLLNAPATVCGPKQSTDLVKNKCVNF
BM48-31/BGR/2008 QSSGIGFQPYRVVVLSFELLNAPATVCGPKQSTELVKNKCVNF
BatCoV/BB9904/BGR/2008 QSSGISFQPYRVVVLSFELLNAPATVCGPKQSTELVKDKCVNF
Civet TTSGIGYQPYRVVVLSFELLNAPATVCGPKLSTDLIKNQCVNF
Pangolin PTTGVNYQPFRVVVLSFELLNGPATVCGPKLSTTLVKDKCVNF
RaTG13 PTDGVGHQPYRVVVLSFELLNAPATVCGPKKSTNLVKNKCVNF
RhBetaCoV_Murcia2022 PTSGVSYQPFRVVVLSFELLNAPATVCGPKQSTDLVKNKCVNF
BtKY72 PTVGVGYQPYRVVVLSFELLNAPATVCGPKKSTELVKNKCVNF
Khosta-2 STSGVGYQPFRVVVLSFELLNAPATVCGPKQSTDLVKNKCVNF
WIV1 ITNGIGYQPYRVVVLSFELLNAPATVCGPKLSTDLIKNQCVNF
WIV16 ITNGIGYQPYRVVVLSFELLNAPATVCGPKLSTDLIKNQCVNF
RsSHC014 TTAGVGHQPYRVVVLSFELLNAPATVCGPKLSTDLIKNQCVNF
Rs7327 TTNGIGYQPYRVVVLSFELLNAPATVCGPKLSTDLIKNQCVNF
HKU3-1 PNVPVAYQATRVVVLSFELLNAPATVCGPKLSTELVKNQCVNF
Rf4092 PNVPLDYQATRVVVLSFELLNAPATVCGPKLSTQLVKNQCVNF
Rs4237 PTVPIEYQATRVVVLSFELLNAPATVCGPKLSTGLVKNQCVNF
Rs4247 PNVPVAYQATRVVVLSFELLNAPATVCGPKLSTQLVKNQCVNF
