# Supplementary material for: Coronavirus Diversity in an Italian Bat Population Sampled During the Pandemic in 2020
Source: Animals (Basel). 2026 Oct 21;16(20):3319. doi: 10.3390/ani16203319 (PMC13700689; doi:10.3390/ani16203319)
Supplement: Supplementary file 1 [file animals-16-03319-s001.zip › Suppl_Fig_captions_Animals.pdf]

Figure captions

Supplementary Figure S1. Phylogenetic tree built using Alpha-CoV complete genomes (Dataset A). The maximum likelihood tree was built using the GTR+F+I+G model based on 1,000 bootstrap replications, with bootstrap values of >70 indicated at their respective nodes.

Supplementary Figure S2. Phylogenetic tree built using Beta-CoV complete genomes (Dataset B). The maximum likelihood tree was built using the GTR+F+I+G model based on 1,000 bootstrap replications, with bootstrap values of >70 indicated at their respective nodes.

Supplementary Figure S3. Alignment of amino acid sequences and secondary structure of Sarbecovirus RBD protein sequences.
