# Supplementary material for: Coronavirus Diversity in an Italian Bat Population Sampled During the Pandemic in 2020
Source: Animals (Basel). 2026 Oct 21;16(20):3319. doi: 10.3390/ani16203319 (PMC13700689; doi:10.3390/ani16203319)
Supplement: Supplementary file 1 [file animals-16-03319-s001.zip › SupplementaryTable_S1.pdf]

Supplementary Table S1. Overview of bat roosts sampled in this study.

Roost ID	N. fecal samples	Species	Municipality	Province	Region	Collection Date	Estimated colony size	CoVs presence
**MIR**	7	*Myotis emarginatus*	Mirano	Venezia	Veneto	19/06/2020	12	0
**OS**	8	*Miniopterus schreibersii* *Myotis myotis* *Myotis blythii*	Osoppo	Udine	Friuli Venezia Giulia	22/06/2020	50-70	1
**PIN**	9	*Rhinolophus hipposideros*	Pinzano al Tagliamento	Pordenone	Friuli Venezia Giulia	23/06/2020	70-100	1
**RIV**	2	*Myotis daubentonii*	Rivoli Veronese	Verona	Veneto	23/06/2020	40-50	0
**VIT1**	4	*Rhinolophus hipposideros*	Vittorio Veneto	Treviso	Veneto	26/06/2020	10	1
**VIT2**	7	*Rhinolophus hipposideros*	Vittorio Veneto	Treviso	Veneto	26/06/2020	10	1
