# Supplementary material for: Coronavirus Diversity in an Italian Bat Population Sampled During the Pandemic in 2020
Source: Animals (Basel). 2026 Oct 21;16(20):3319. doi: 10.3390/ani16203319 (PMC13700689; doi:10.3390/ani16203319)
Supplement: Supplementary file 1 [file animals-16-03319-s001.zip › SupplFigS1.pdf]

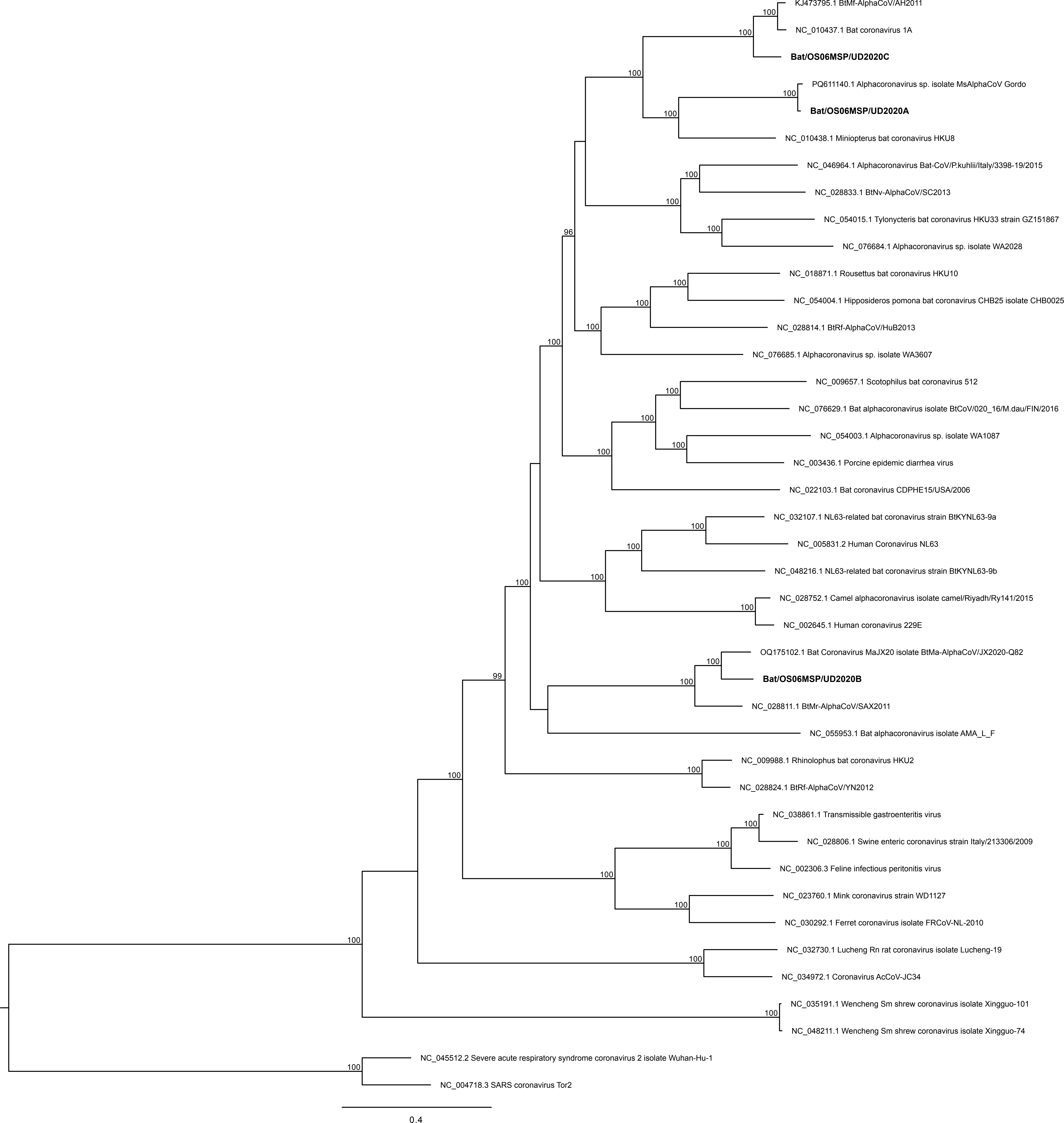

KJ473795.1 BtMf-AlphaCoV/AH2011
NC_010437.1 Bat coronavirus 1A
Bat/OS06MSP/UD2020C
PQ611140.1 Alphacoronavirus sp. isolate MsAlphaCoV Gordo
Bat/OS06MSP/UD2020A
NC_010438.1 Miniopterus bat coronavirus HKU8
NC_046964.1 Alphacoronavirus Bat-CoV/P.kuhlii/Italy/3398-19/2015
NC_028833.1 BtNv-AlphaCoV/SC2013
NC_054015.1 Tylonycteris bat coronavirus HKU33 strain GZ151867
NC_076684.1 Alphacoronavirus sp. isolate WA2028
NC_018871.1 Rousettus bat coronavirus HKU10
NC_054004.1 Hipposideros pomona bat coronavirus CHB25 isolate CHB0025
NC_028814.1 BtRf-AlphaCoV/HuB2013
NC_076685.1 Alphacoronavirus sp. isolate WA3607
NC_009657.1 Scotophilus bat coronavirus 512
NC_076629.1 Bat alphacoronavirus isolate BtCoV/020_16/M.dau/FIN/2016
NC_054003.1 Alphacoronavirus sp. isolate WA1087
NC_003436.1 Porcine epidemic diarrhea virus
NC_022103.1 Bat coronavirus CDPHE15/USA/2006
NC_032107.1 NL63-related bat coronavirus strain BtKYNL63-9a
NC_005831.2 Human Coronavirus NL63
NC_048216.1 NL63-related bat coronavirus strain BtKYNL63-9b
NC_028752.1 Camel alphacoronavirus isolate camel/Riyadh/Ry141/2015
NC_002645.1 Human coronavirus 229E
OQ175102.1 Bat Coronavirus MaJX20 isolate BtMa-AlphaCoV/JX2020-Q82
Bat/OS06MSP/UD2020B
NC_028811.1 BtMr-AlphaCoV/SAX2011
NC_055953.1 Bat alphacoronavirus isolate AMA_L_F
NC_009988.1 Rhinolophus bat coronavirus HKU2
NC_028824.1 BtRf-AlphaCoV/YN2012
NC_038861.1 Transmissible gastroenteritis virus
NC_028806.1 Swine enteric coronavirus strain Italy/213306/2009
NC_002306.3 Feline infectious peritonitis virus
NC_023760.1 Mink coronavirus strain WD1127
NC_030292.1 Ferret coronavirus isolate FRCoV-NL-2010
NC_032730.1 Lucheng Rn rat coronavirus isolate Lucheng-19
NC_034972.1 Coronavirus AcCoV-JC34
NC_035191.1 Wencheng Sm shrew coronavirus isolate Xingguo-101
NC_048211.1 Wencheng Sm shrew coronavirus isolate Xingguo-74
NC_045512.2 Severe acute respiratory syndrome coronavirus 2 isolate Wuhan-Hu-1
NC_004718.3 SARS coronavirus Tor2
0.4
