# Supplementary material for: Coronavirus Diversity in an Italian Bat Population Sampled During the Pandemic in 2020
Source: Animals (Basel). 2026 Oct 21;16(20):3319. doi: 10.3390/ani16203319 (PMC13700689; doi:10.3390/ani16203319)
Supplement: Supplementary file 1 [file animals-16-03319-s001.zip › SupplFigS2.pdf]

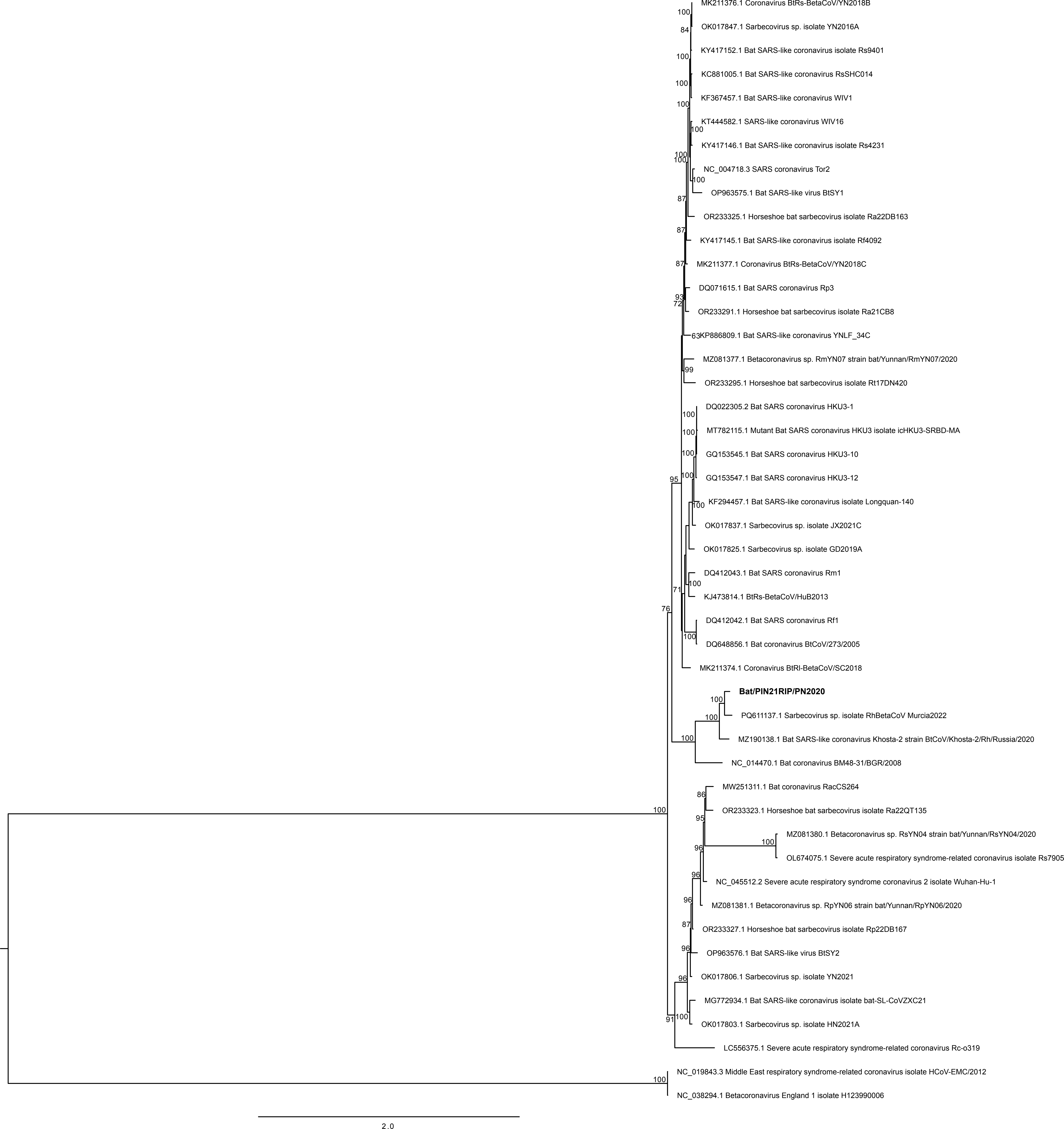
MK211376.1 Coronavirus BtRs-BetaCoV/YN2018B
OK017847.1 Sarbecovirus sp. isolate YN2016A
KY417152.1 Bat SARS-like coronavirus isolate Rs9401
KC881005.1 Bat SARS-like coronavirus RsSHC014
KF367457.1 Bat SARS-like coronavirus WIV1
KT444582.1 SARS-like coronavirus WIV16
KY417146.1 Bat SARS-like coronavirus isolate Rs4231
NC_004718.3 SARS coronavirus Tor2
OP963575.1 Bat SARS-like virus BtSY1
OR233325.1 Horseshoe bat sarbecovirus isolate Ra22DB163
KY417145.1 Bat SARS-like coronavirus isolate Rf4092
MK211377.1 Coronavirus BtRs-BetaCoV/YN2018C
DQ071615.1 Bat SARS coronavirus Rp3
OR233291.1 Horseshoe bat sarbecovirus isolate Ra21CB8
KP886809.1 Bat SARS-like coronavirus YNLF_34C
MZ081377.1 Betacoronavirus sp. RmYN07 strain bat/Yunnan/RmYN07/2020
OR233295.1 Horseshoe bat sarbecovirus isolate Rt17DN420
DQ022305.2 Bat SARS coronavirus HKU3-1
MT782115.1 Mutant Bat SARS coronavirus HKU3 isolate icHKU3-SRBD-MA
GQ153545.1 Bat SARS coronavirus HKU3-10
GQ153547.1 Bat SARS coronavirus HKU3-12
KF294457.1 Bat SARS-like coronavirus isolate Longquan-140
OK017837.1 Sarbecovirus sp. isolate JX2021C
OK017825.1 Sarbecovirus sp. isolate GD2019A
DQ412043.1 Bat SARS coronavirus Rm1
KJ473814.1 BtRs-BetaCoV/HuB2013
DQ412042.1 Bat SARS coronavirus Rf1
DQ648856.1 Bat coronavirus BtCoV/273/2005
MK211374.1 Coronavirus BtRl-BetaCoV/SC2018
Bat/PIN21RIP/PN2020
PQ611137.1 Sarbecovirus sp. isolate RhBetaCoV Murcia2022
MZ190138.1 Bat SARS-like coronavirus Khosta-2 strain BtCoV/Khosta-2/Rh/Russia/2020
NC_014470.1 Bat coronavirus BM48-31/BGR/2008
MW251311.1 Bat coronavirus RacCS264
OR233323.1 Horseshoe bat sarbecovirus isolate Ra22QT135
MZ081380.1 Betacoronavirus sp. RsYN04 strain bat/Yunnan/RsYN04/2020
OL674075.1 Severe acute respiratory syndrome-related coronavirus isolate Rs7905
NC_045512.2 Severe acute respiratory syndrome coronavirus 2 isolate Wuhan-Hu-1
MZ081381.1 Betacoronavirus sp. RpYN06 strain bat/Yunnan/RpYN06/2020
OR233327.1 Horseshoe bat sarbecovirus isolate Rp22DB167
OP963576.1 Bat SARS-like virus BtSY2
OK017806.1 Sarbecovirus sp. isolate YN2021
MG772934.1 Bat SARS-like coronavirus isolate bat-SL-CoVZXC21
OK017803.1 Sarbecovirus sp. isolate HN2021A
LC556375.1 Severe acute respiratory syndrome-related coronavirus Rc-o319
NC_019843.3 Middle East respiratory syndrome-related coronavirus isolate HCoV-EMC/2012
NC_038294.1 Betacoronavirus England 1 isolate H123990006
2.0
